# Dental Caries Status in Postmenopausal Women: Systematic Review and Meta-Analysis

**DOI:** 10.3390/jcm14061837

**Published:** 2025-03-08

**Authors:** Victoria Areal-Quecuty, Juan J. Segura-Egea, Aurea Simón-Soro, María León-López, Cristiane Cantiga-Silva, Jenifer Martín-González, Benito Sánchez-Domínguez, Daniel Cabanillas-Balsera

**Affiliations:** 1 Endodontic Section, Department of Stomatology, School of Dentistry, University of Sevilla, C/Avicena s/n, 41009 Sevilla, Spain; vareal@us.es (V.A.-Q.); asimon@us.es (A.S.-S.); maria.leon.lopez.98@gmail.com (M.L.-L.); jmartin30@us.es (J.M.-G.); 2 Department of Preventive and Restorative Dentistry, School of Dentistry, Säo Paulo State University (UNESP), Araçatuba 16015-050, SP, Brazil; cristiane.cantiga@gmail.com; 3 Andalusian Health Service, 41071 Sevilla, Spain; beni2506@yahoo.es

**Keywords:** dental caries, menopause, meta-analysis, older adults, postmenopausal women, systematic review

## Abstract

**Background**: Dental caries is one of the most common oral infections observed worldwide. It is defined as a multifactorial dynamic disease-causing mineral loss of dental hard tissue, which is identified by the caries lesion. Treatment of the caries lesion involves filling the cavity or removing the damaged tooth. Then, the decayed, missing, and filled teeth (DMFT) index is the simplest and most commonly used index to assess the dental caries status. Salivary glands are estrogen dependent and, after menopause, the changes in salivary flow and saliva consistency produce xerostomia, hyposiale, or dryness, common findings among postmenopausal women. Since saliva plays a fundamental role in caries prevention, the postmenopausal decline in salivary secretion may contribute to increased caries incidence. The aim of this systematic review and meta-analysis was to answer the following PICO question: In adult women (P), does the presence of menopause (I), compared to its absence (C), influence dental caries status, assessed using the DMFT index (O)? **Methods**: The study adhered to PRISMA guidelines. A systematic search was conducted in PubMed/MEDLINE, Scopus, and EMBASE databases. For each study, characteristics and mean difference (MD) with 95% CI were extracted. Meta-analyses were performed using the Revman software (v. 5.4) to calculate pooled MD and 95% CI. Random-effects model meta-analysis was performed. Risk of bias was evaluated using the Newcastle–Ottawa Scale adapted for cross-sectional studies. To estimate variance and heterogeneity between trials, the Higgins I^2^ test was used. The certainty level of the evidence was determined through the GRADE approach. **Results**: Seven studies fulfilled the inclusion criteria, including 4396 postmenopausal women and 5131 control women. Meta-analysis showed an overall MD = 3.13 (95% CI = 2.12–4.15; *p* < 0.00001), which suggest that postmenopausal women had a DMFT index 3 units higher than the control group. **Conclusions**: Menopause was significantly associated with worse dental caries status, probably due to declining estrogen levels affecting salivary function. Further research is needed to confirm mechanisms and evaluate preventive strategies like hormone replacement therapy.

## 1. Introduction

Estrogens are steroid hormones considered essential for maintaining homeostasis and systemic health. Its role in developing and maintaining reproductive tissues is widely known. Furthermore, estrogens have many beneficial effects on several nonreproductive target tissues, such as liver, adipose tissue, bone, skeletal muscle, and blood vessels [1]. Estrogen imbalances, especially the decrease in estrogen that occurs after menopause, can alter systemic and oral health, causing osteoporosis [2], burning mouth, and changes in palate perception [3]. Moreover, postmenopausal estrogen deficiency can lead to development of gingivitis, periodontitis, and osteoporotic jaws [4,5].

One of the main oral effects of estrogen deficiency is the change in the quantity and quality of saliva [6]. Salivary glands are estrogen dependent and, after menopause, the changes in salivary flow and saliva consistency produce xerostomia, hyposiale, or dryness, common findings among postmenopausal women [7]. Taking into account that saliva plays a fundamental role in defending against caries, the decrease in salivary secretion that occurs after menopause could increase the incidence of caries lesions [4].

Dental caries is one of the most common oral infections observed worldwide [8]. It is defined as a multifactorial dynamic disease-causing mineral loss of dental hard tissue [9]. Caries is a significant public health problem for population and governments worldwide [10]. Treatment of the caries lesion involves filling the cavity or removing the damaged tooth. Then, the decayed, missing, and filled teeth (DMFT) index is the simplest and most commonly used index to assess the dental caries status. DMFT is the sum of the number of decayed, missing due to caries, and filled teeth in the permanent dentition. Therefore, the DMFT index ranges from 0 to 28 (excluding third molars), where higher scores indicate poorer dental health due to more decayed, missing, or filled teeth. This index is widely used in epidemiological studies to assess oral health. Some studies have found an association between menopause and the prevalence of caries lesions, with mixed results [11,12]. Moreover, the results of a recently published systematic review suggest the existence of a significant association between the prevalence of chronic apical periodontitis, a disease directly related to caries, and menopause [13].

The aim of this systematic review and meta-analysis was to analyze the scientific literature about the association between menopause and caries dental status, assessed using the DMFT index. The research question was: In adult women, does the presence or absence of menopause influence dental caries status, assessed using the DMFT index?

## 2. Materials and Methods

This systematic review complies with the guidelines outlined by the Preferred Reporting Items for Systematic Reviews and Meta-Analyses (PRISMA) [14]. This systematic review has been submitted to PROSPERO (655622).

### 2.1. Review Question

The research question was structured using the PICO method: In adult women (Population), does the presence of menopause (Intervention/Condition), compared to its absence (Comparison), influence dental caries status, assessed using the DMFT index (Outcome)? A thorough literature search was conducted to identify the articles related to dental caries status, comparing postmenopausal women with healthy control women.

### 2.2. Eligibility Criteria

Inclusion criteria were established as (a) clinical epidemiological studies published between January 1980 and December 2024; (b) studies comparing postmenopausal women to healthy premenopausal women; and (c) studies providing dental caries status using the DMFT index, both in control and postmenopausal women.

The exclusion criteria were as follows: (a) studies conducted on animals or in vitro; (b) case series; (c) studies reporting data exclusively on menopausal women; and (d) studies not reporting DMFT index.

### 2.3. Search Strategy and Information Sources

A literature search was undertaken with no limits on time or language until December 2024 in the PubMed-MEDLINE, SCOPUS, and EMBASE databases, utilizing a combination of Medical Subject Headings (MeSH) and relevant keywords. The search strategy incorporated terms such as: *(“caries”[All Fields] OR “dental caries”[MeSH Terms] OR (“dental”[All Fields] AND “caries”[All Fields]) OR “dental caries”[All Fields] OR “caries”[All Fields] OR “DMFT”[All Fields]) AND (“estrogen s”[All Fields] OR “estrogene”[All Fields] OR “estrogenes”[All Fields] OR “estrogenic”[All Fields] OR “estrogenically”[All Fields] OR “estrogenicities”[All Fields] OR “estrogenicity”[All Fields] OR “estrogenization”[All Fields] OR “estrogenized”[All Fields] OR “oestrogen”[All Fields] OR “estrogens”[Pharmacological Action] OR “estrogens”[MeSH Terms] OR “estrogens”[All Fields] OR “estrogen”[All Fields] OR “oestrogens”[All Fields] OR “oestrogenic”[All Fields] OR “oestrogenically”[All Fields] OR “oestrogenicity”[All Fields] OR “oestrogenization”[All Fields] OR “oestrogens”[All Fields] OR (“menopause”[MeSH Terms] OR “menopause”[All Fields] OR “menopausal”[All Fields] OR “menopaused”[All Fields] OR “menopauses”[All Fields]) OR (“postmenopausal”[All Fields] OR “postmenopausally”[All Fields] OR “postmenopausals”[All Fields] OR “postmenopause”[MeSH Terms] OR “postmenopause”[All Fields] OR “postmenopausic”[All Fields])).*

A complementary screening on the references of the selected studies was performed to find any additional study that did not appear in the primary database search. Gray literature was searched (https://opengrey.eu/; https://scholar.google.com/; https://www.greynet.org/) but did not provide useful data.

Two independent reviewers (J.J.S.-E. and V.A.-Q.) conducted the literature search and screened articles for eligibility. Initially, the titles and abstracts were reviewed to assess their relevance to the review. Subsequently, a thorough analysis of the full texts was carried out, evaluating them based on the pre-established inclusion and exclusion criteria. In the event of any discrepancies between the reviewers, these were resolved through direct discussion or, when necessary, by consulting a third reviewer (D.C.-B.), in order to ensure consistency and accuracy in the article-selection process.

### 2.4. Data Extraction

One author (V.A.-Q.) collected the information of the studies that matched the inclusion criteria individually, while other three authors (C.C.-S., D.C.-B. and J.J.S.-E.) verified the inclusion criteria and tabulated data to ensure the absence of errors. Disagreements were resolved by discussion and consensus. For each study, key methodological details were extracted, such as author information, publication year, study design, sample characteristics, quantitative findings, mean differences, and the method used to diagnose caries lesions. Dental caries diagnosed using the DMFT index was the primary outcome measure. The authors of the original studies were contacted for clarification if necessary.

### 2.5. Data Synthesis and Analysis

The outcome variable studied was dental caries status, calculated using the DMFT index. In each included study, the MD in the DMFT index was calculated with its 95% confidence interval (CI), aiming to measure the effect of the relationship between menopause and dental caries status.

A random-effects model meta-analysis was performed using Revman software (version 5.4) to calculate pooled mean difference (MD) and 95% confidence interval (CI).

The primary analysis focused on the dental caries status in postmenopausal versus premenopausal healthy control women. Forest plots illustrated the MD across studies. Statistical significance was set at *p* = 0.05. Heterogeneity among studies was assessed using Higgins’ I^2^ statistic, with thresholds of 25–50% for low heterogeneity, 50–75% for moderate heterogeneity, and >75% for high heterogeneity [15].

### 2.6. Risk of Bias Assessment

The risk of bias in the included studies was evaluated using the Newcastle–Ottawa Scale [16], adapted for cross-sectional research [17]. The assessment focused on two domains adapted to the outcome of interest: sample selection and outcome. Points (*) were allocated based on the presence or absence of key criteria. Three researchers (V.A.-Q., D.C.-B., and J.J.S.-E.) independently assessed the studies, resolving any disagreements through discussion. The evaluation of each item was made according to the following criteria:

(A) Sample Selection Domain (maximum: 6 points):
  (1) Sample representativeness: random sampling (3 points), non-random sampling (2 points), selected groups (1 point), or no description (0 points).
  (2) Sample size: justified by calculations or entire population recruitment with ≤20% loss (1 point); otherwise, (0 points).
  (3) Menopausal status confirmation: cessation of menstruation (2 point); only by age (1 point); no criteria description (0 points).
(B) Outcome Domain (maximum: 3 points):
  (1) Outcome assessment: DMFT index registered with inter- and intra-examiner reliability reported (2 points), partial reliability (1 point), or none (0 points).
  (2) Number of observers: Two or more examiners (1 point); otherwise (0 points).

Studies could score a maximum of 9 points. They were defined as high risk of bias if they scored 0–3 points, moderate risk of bias if they scored 4–6 points, and low risk of bias if they scored 7–9 points.

### 2.7. Grading of Recommendations Assessment, Development, and Evaluation

The Grading of Recommendations Assessment, Development, and Evaluation (GRADE) framework was used to evaluate the overall quality and certainty of the evidence [18,19]. This process was independently conducted by three researchers (V.A.-Q., J.J.S.-E. and D.C.-B.). The GRADE tool is used to systematically assess the quality of evidence on health research. It provides a transparent framework for evaluating the certainty of evidence and for making recommendations based on this evidence. The GRADE approach considers the study design, the consistency of results across studies, the directness of the evidence, the heterogeneity of the studies, the imprecision, and the potential biases, such as risk of bias and publication bias.

The tool helps guide healthcare professionals and policymakers in making informed decisions by clearly defining the strength of recommendations according to the quality of available evidence. The GRADE system categorizes evidence into four levels—high, moderate, low, or very low—based on the certainty of the estimates, influencing the confidence in clinical decisions [20].

## 3. Results

**Figure 1:**
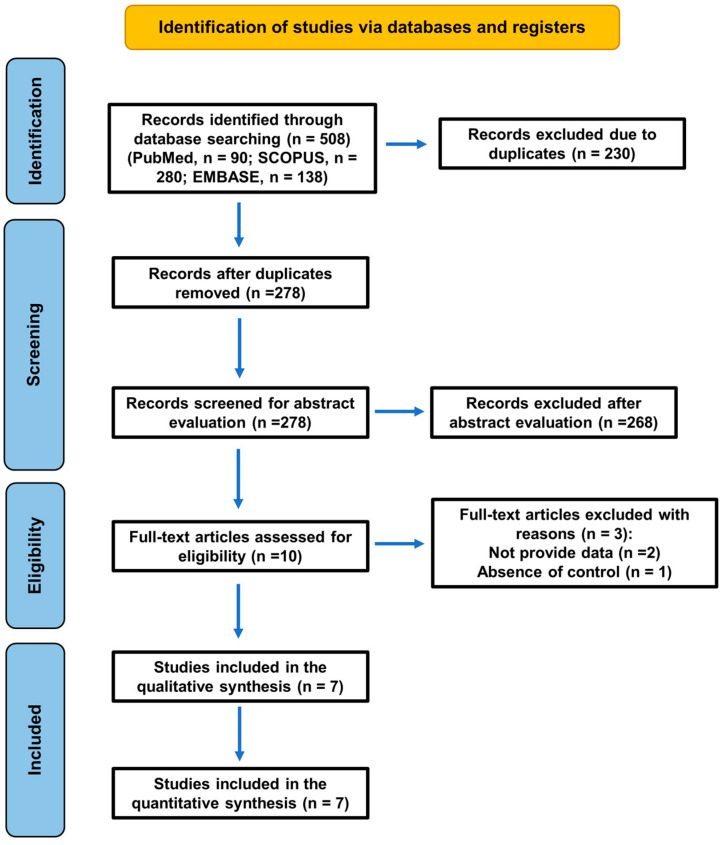
A flowchart of the search strategy following the PRISMA 2020 guidelines for systematic reviews and meta-analyses.

The flow diagram of the literature search strategy and selected studies from databases and registers is shown in Figure 1, in accordance with PRISMA 2020 instructions [14,21].

A total of 508 records (PubMed, *n* = 90; SCOPUS, *n* = 280; EMBASE, *n* = 138) were initially identified, but 230 were duplicates. Two hundred and seventy-eight records were screened for abstract evaluation, and two hundred and sixty-eight were excluded for not meeting the inclusion criteria. During the eligibility assessment, 3 reports were excluded for two specific reasons: lack of quantitative data (not reporting the necessary numerical values) [7,22] and absence of healthy controls [23] (Table 1).

### 3.1. Characteristics of the Included Studies

Finally, seven studies [11,12,24,25,26,27,28] were deemed eligible and included in the analysis. The descriptive characteristics of the studies included in the analysis are represented in Table 2.

The table includes details on the authors and publication years, study designs, sample sizes for control and menopausal groups, and the main results regarding the association. All studies employed a cross-sectional design and involved varying sample sizes, ranging from small groups with 14 participants [28] to much larger cohorts, including over 5000 participants in the control group [12]. Most studies reported a statistically significant association (*p* < 0.05) between menopause and increased caries risk, except for the study by Foglio-Bonda et al. [11], which found no significant relationship (*p* > 0.05).

Table 3 presents the data extraction, comparing the DMFT index between individuals with and without estrogen deficiency across selected studies. The seven studies included a total of 4396 menopausal patients and 5131 non-menopausal control subjects.

The majority of the studies demonstrate a higher DMFT index among estrogen-deficient patients compared to non-deficient controls, indicating a potential negative impact of estrogen deficiency on oral health. Lee et al. [12] reported a mean DMFT index of 8.83 in the estrogen-deficient group versus 6.62 in controls, while Dural et al. [27] found an even greater disparity, with indices of 13.2 and 5.7, respectively. Standard deviations across studies reveal variability within groups, but the overall trend consistently favors worse dental outcomes in the estrogen-deficient population.

### 3.2. Meta-Analysis of the Prevalence of Caries Assessed Using the DMFT Index

**Figure 2:**
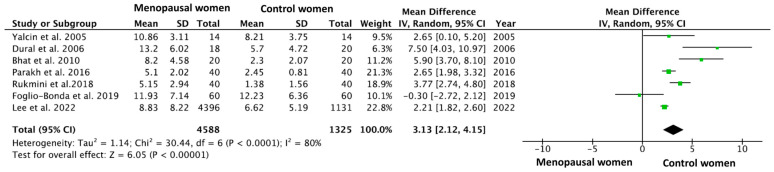
A forest plot of the mean difference and its 95% confidence interval comparing the prevalence of apical periodontitis in patients with arteriosclerosis and healthy control patients. The estimate is based on data from the seven selected studies [11,12,24,25,26,27,28].

The meta-analysis was performed using data from Table 3, and its forest plot is shown in Figure 2. The forest plot summarized mean differences (MD) and confidence intervals (CI) across included studies, providing a visual representation of the pooled effect size and its statistical significance. The overall MD was calculated using a random-effects model meta-analysis, resulting in a MD = 3.13 (95% CI = 2.12–4.15; *p* < 0.00001), which means that postmenopausal women had a DMFT index 3 units higher than the control patients.

All the studies included in the meta-analysis reported a higher DMFT index in postmenopausal patients, except for the study by Foglio-Bonda et al., 2019 [11], which indicated a higher index in the control group (11.93 in the estrogen-deficient group versus 12.23 in control group). Still, the forest plot shows a strong statistical significance in the results (*p* < 0.00001). The heterogeneity value was I^2^ = 80%.

### 3.3. Risk of Bias Assessment

The risk of bias for each of the individual studies included in the review was assessed using the Newcastle–Ottawa Scale [16], with a maximum possible score of 9 points, represented in Table 4. A higher score indicates a lower risk of bias. The studies were categorized into three groups based on their scores: high risk (0 to 3 points), moderate risk (4 to 6 points), and low risk (7 to 9 points). All studies included in this systematic review with meta-analysis obtained a moderate risk of bias, except for the article by Foglio-Bonda et al., 2019 [11], which was classified as having a high risk of bias.

### 3.4. Publication Bias

**Figure 3:**
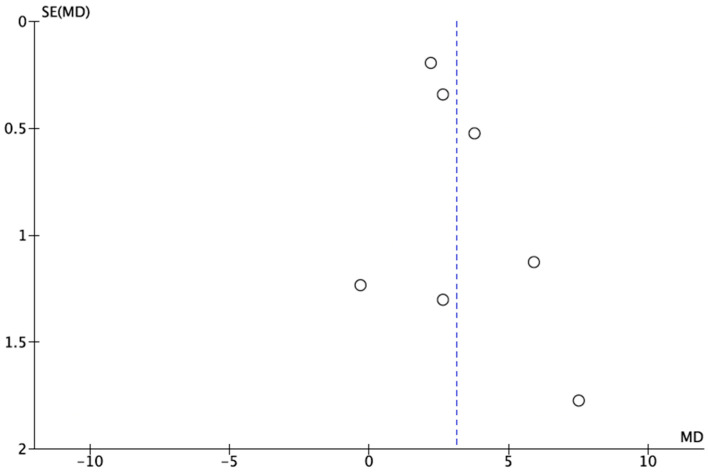
A funnel plot for estimates in meta-analysis of the dental caries status in postmenopausal women and healthy control patients. Circles represent included studies and dotted line represents overall effect. Studies with higher power and lower standard error are placed towards the top. Studies with lower power are placed towards the bottom.

It was not possible to quantitatively assess publication bias due to the inclusion of fewer than the minimum required 10 studies [15]. However, a funnel plot was plotted to illustrate the possible existence of publication bias (Figure 3).

### 3.5. GRADE Evaluation: Level of Certainty

Table 5 presents the GRADE assessment of the certainty level regarding the association between menopause and the prevalence of dental caries. A high degree of inconsistency was observed, with an I^2^ value of 80% (*p* < 0.0001), indicating significant heterogeneity among the studies.

The inclusion of studies with limited representation of diverse populations restricts the generalizability of the results to broader demographic groups. Therefore, indirectness has been rated as serious. Imprecision was rated as serious due to the small sample sizes in subgroup analyses. The estimated effect size, represented by a MD of 3.13 (95% CI: 2.12–4.15), suggests a substantial association between menopause and increased dental caries prevalence. However, as all five included studies are cross-sectional, the level of certainty was downgraded to very low, implying that the actual effect is likely to be substantially different from what the evidence suggests. Thus, recommendations based on this evidence should be made with caution.

## 4. Discussion

This systematic review and meta-analysis aimed to analyze the available evidence about the association between menopause and the risk of dental caries. The meta-analysis showed that postmenopausal women have worse dental caries status than the control group with a greatly significant *p* value (MD = 3.13; 95% CI = 2.12–4.15; *p* < 0.00001).

The overall result suggest that postmenopausal women have significantly higher DMFT index values than the control patients, meaning they have more decayed, missing or filled teeth on average. In practical terms, the DMFT index of postmenopausal women is 3.13 units higher compared to the control group. This difference in means is considered large [29].

This finding is relevant because it could imply that postmenopausal women are at a higher risk of dental problems compared to the control group, which may be related to hormonal or other changes associated with menopause.

### 4.1. Risk of Bias of Included Studies

Regarding the risk of bias of the included studies, the study by Lee et al. [12] was classified as having a moderate risk of bias, but it received a low-risk rating in the sample representativeness section, as it was the only study among all the included ones that conducted random sampling in its methodology. These studies exhibited limitations in areas such as sample selection and outcome assessment, which contributed to their scores. The study by Rukmini et al. [24] was also classified as having a moderate risk of bias, but it was the only one of all the included studies that performed a sample-size calculation in its research, which reduces the risk of bias in its methodology and allows for a more confident generalization of its results to the overall population. The study by Foglio-Bonda et al. [11] exhibits a high risk of bias due to minimal representativeness and insufficient attention to sample size and outcome assessment. Across all studies, the absence of multiple observers and inconsistencies in sample representativeness emerge as critical sources of bias, indicating potential limitations in the reliability of the reported outcomes.

### 4.2. The Heterogeneity of the Included Studies

This meta-analysis underscores the association between estrogen deficiency and increased dental disease risk, while also highlighting the need to account for heterogeneity in sample sizes, methodologies, and population characteristics when interpreting the results. Such heterogeneity (I^2^ = 80%; *p* < 0.0001), emphasizes the importance of cautious generalization and the potential need for further research to confirm these findings. The final MD, greater than 3, reflects a statistically significant association between menopause and an increased risk of dental caries or worse oral health.

### 4.3. Menopause as a Risk Factor for Caries and Other Oral Diseases

Over the last decades, several studies have established a disparity between the genders and the risk of developing caries; females seem to have a higher prevalence of carious lesions compared to males [30,31,32]. Altered estrogen levels have been related to woman’s worse oral health, including dental caries [32]. In fact, a study of caries patterns in medieval London established that caries frequency and sex are significantly associated, showing an increased prevalence of caries in females [33].

The most frequent oral symptom reported in menopausal women is oral dryness [4,28]. A reduction in salivary secretion can lead to several oral problems. Saliva is considered to be the principal defense factor in the oral cavity [24]. The insufficiency of saliva has been described as one of the causes of an upsurge in dental caries [4].

Among other factors, the salivary flow has a caries-preventive effect by modulating sugar’s dilution or elimination process. Therefore, patients with a higher flow rate have a faster clearance rate and a low incidence of caries [34].

The relationship between menopausal status and oral health has been extensively explored, with numerous studies highlighting significant deterioration in postmenopausal women [22,28]. Several investigations have consistently reported a worse DMFT index in postmenopausal women compared to premenopausal controls, which has been attributed to factors such as reduced salivary flow rate, lower salivary pH, and poor oral hygiene indices [27]. For example, reductions in salivary parameters have been closely linked to increased DMFT index values, emphasizing the importance of salivary protection in oral health [26]. A significant correlation between salivary flow and DMFT has been established, with diminished flow and pH levels observed in postmenopausal women compared to controls, accompanied by higher DMFT values [24,25].

These findings suggest a potential hormonal influence on salivary function and oral health, reinforcing the role of estrogen in maintaining oral homeostasis. As estrogen levels decline with menopause, the protective effect of this hormone on oral tissues appears to wane, leading to increased susceptibility to dental caries. Age-related increases in the DMFT index further support this hypothesis, as salivary gland function and hormonal balance deteriorate over time [7,12].

Interestingly, contrasting results have also been reported. No significant differences in DMFT indices between menopausal and premenopausal groups were found, which may be explained by comparable ages and estrogen levels in the control and study populations. These discrepancies underscore the complex interplay between hormonal status, salivary parameters, and caries prevalence, necessitating further research to clarify these relationships [11].

The potential impact of hormone replacement therapy (HRT) on oral health has been another area of interest [35,36]. Studies suggest that HRT may have a protective effect, with lower caries prevalence observed in hormone users compared to non-users [23]. This finding highlights the potential of hormonal interventions not only for managing menopausal symptoms but also for mitigating oral health deterioration in this population.

Beyond menopause, the role of estrogen in dental caries etiology has been investigated across various age groups. Estrogen is known to play critical roles in numerous tissues, including during amelogenesis [37]. Recent studies have explored genetic polymorphisms in estrogen receptor genes, particularly ERα, and their association with dental conditions [38]. These polymorphisms have also been implicated in other oral health conditions, such as jaw disorders [39]. While certain polymorphisms have been linked to enamel defects, no significant association with caries experience has been established, suggesting that estrogen’s impact on caries may be mediated by factors beyond genetic predisposition [38].

These findings collectively emphasize the multifactorial nature of dental caries in postmenopausal women and highlight the need for an integrative approach to understanding and addressing this issue. Hormonal status, genetic factors, and salivary function all contribute to the observed disparities, providing avenues for targeted interventions and future research.

The clinical implications for postmenopausal women highlight the importance of regular check-ups to monitor salivary flow and oral health. It is crucial to provide education on proper oral hygiene, the impact of menopause on oral health and the consequences of reduced estrogen levels. Recommendations such as saliva substitutes and personalized interventions, including dietary control and salivary pH monitoring, can help mitigate caries risk. Additionally, hormone replacement therapy (HRT) may offer protective benefits in reducing oral health deterioration.

### 4.4. Limitations and Strengths

Although the risk of bias and heterogeneity of the studies included in the systematic review have already been mentioned, it is worth analyzing to what extent these limit the results of the review.

Most studies included in the review were classified as moderate risk of bias. The domain that posed the greatest limitations in the articles was the sample selection. Only one of the seven included studies used random sampling for their research, while the other studies used a selected group of subjects, which is considered a very high risk of bias. Another methodological flaw in the included studies was that only one of the studies in the review calculated and justified the sample size used in the research [24], while the other studies did not perform the necessary sample-size calculation.

This variability in methodological quality is reflected in the GRADE assessment [19], which rated the certainty of evidence as very low. Factors contributing to this rating include a serious risk of bias, indirectness, imprecision due to small subgroup sample sizes, and significant heterogeneity across studies.

Despite these findings, the results must be interpreted with caution due to several limitations inherent to the included studies and the meta-analysis process.

Additionally, publication bias could not be assessed quantitatively, as fewer than ten studies were included in the meta-analysis. This limitation further emphasizes the need for caution when generalizing these findings. The heterogeneity observed in the meta-analysis may be attributed to differences in study designs, population characteristics, and methods used for caries assessment, all of which underscore the necessity for standardized methodologies in future research.

Another important limitation is the reliance on observational studies with varying sample sizes. Discrepancies in sample sizes contribute to inconsistencies in the findings and reduce the precision of the pooled estimates. Moreover, the inclusion of studies with a limited representation of diverse populations restricts the generalizability of the results to broader demographic groups.

Despite a mean difference of 3.13 being statistically significant, its clinical relevance depends on various factors, including baseline oral health status, access to preventive care, and individual susceptibility to caries progression. While this difference may indicate an increased need for dental treatment, its actual impact on patient outcomes should be interpreted in the context of broader oral health determinants.

Demographic differences across study populations, including socioeconomic status, nationality, and oral hygiene habits, may significantly influence the observed outcomes and contribute to the disparities among studies, even when analyzing populations of similar ages. For instance, the notable difference in DMFT scores between the control groups in the studies by Foglio-Bonda et al. [11] and Bhat et al. [26] could be attributed to variations in access to dental care, dietary patterns, and public health policies in their respective regions. Socioeconomic factors play a crucial role in determining the availability and utilization of preventive dental services, while cultural and national differences may influence oral health behaviors, such as frequency of dental visits and fluoride exposure. These discrepancies highlight the complexity of comparing findings across diverse populations and underscore the need for cautious interpretation of pooled data in systematic reviews and meta-analyses.

This study highlights a significant association between menopause and increased risk of dental caries, potentially linked to estrogen deficiency. However, the very low certainty of evidence, heterogeneity, and the limitations of the included studies underscore the need for further research using prospective cohort designs, larger sample sizes, and standardized methodologies. Future studies should also aim to investigate the underlying mechanisms through which hormonal changes during menopause influence oral health, which could guide targeted interventions for this population.

## 5. Conclusions

This systematic review and meta-analysis suggest that postmenopausal women have a worse dental caries status compared to premenopausal healthy control women. While most studies support this relationship, the methodological heterogeneity and cross-sectional design limit the strength of causal inferences. These findings highlight the multifactorial nature of dental caries in postmenopausal women and underscore the need for longitudinal studies to clarify mechanisms and evaluate interventions, including hormone replacement therapy, to mitigate oral health deterioration in this population. It is important for the healthcare community to be aware of this relationship in order to ensure oral care and the prevention of cavities through the promotion of oral hygiene education in postmenopausal patients.

## Figures and Tables

**Table 1:** Excluded studies and the reasons for exclusion.

Reasons	Excluded Studies
Quantitative data not reported	Baum et al., 1981 [22] Mishra et al., 2021 [7]
Absence of control group	Yalcin et al., 2006 [23]

**Table 2:** The descriptive characteristics of the included studies: authors and year, study design, subjects and sample size, and the main results on the association between menopause and caries.

Author	Reference	Study Design	Subjects	Menopause–Caries Association
Lee et al., 2022	[12]	Cross-sectional	Control—5131 Menopause—4396	Yes *p* < 0.05
Foglio-Bonda et al., 2019	[11]	Cross-sectional	Control—60 Menopause—60	No *p* > 0.05
Rukmini et al., 2018	[24]	Cross-sectional	Control—40 Menopause—60	Yes *p* < 0.05
Parakh et al., 2018	[25]	Cross-sectional	Control—40 Menopause—40	Yes *p* < 0.05
Bhat et al., 2010	[26]	Cross-sectional	Control—20 Menopause—20	Yes *p* < 0.05
Dural et al., 2006	[27]	Cross-sectional	Control—20 Menopause—18	Yes *p* < 0.05
Yalcin et al., 2005	[28]	Cross-sectional	Control—14 Menopause—14	Yes *p* < 0.05

**Table 3:** Comparison of the decayed, missing, and filled teeth index (DMFT) between control women and postmenopausal women. The table includes data on authors and year, total sample size, number of subjects, DMFT index (mean and standard deviation) in control women and postmenopausal women, means differences (MD) with 95% CI, and *p* values.

Authors	Sample Size	Control Women		Postmenopausal Women		MD (95% C.I.)	*p*
		DMFT Index	Subjects	DMFT Index	Subjects		
Lee et al., 2022 [12]	9527	6.62 ± 5.19	5131	8.83 ± 8.22	4396	2.21 (1.82–2.6)	<0.05
Foglio-Bonda et al., 2019 [11]	120	12.23 ± 6.36	60	11.93 ± 7.14	60	−0.3 (−2.72–2.12)	>0.05
Rukmini et al., 2018 [24]	80	1.38 ± 1.56	40	5.15 ± 2.94	40	3.77 (2.74–4.8)	<0.05
Parakh et al., 2016 [25]	80	2.45 ± 0.81	40	5.10 ± 2.02	40	2.65 (1.98–3.32)	<0.05
Bhat et al., 2010 [26]	40	2.30 ± 2.07	20	8.20 ± 4.58	20	5.9 (3.7–8.1)	<0.05
Dural et al., 2006 [27]	38	5.7 ± 4.72	20	13.2 ± 6.02	18	7.5 (4.03–10.97)	<0.05
Yalcin et al., 2005 [28]	28	8.21 ± 3.75	14	10.86 ± 3.11	14	2.65 (0.1–5.2)	<0.05

MD: means differences (postmenopausal—control); CI: confidence interval.

**Table 4:** The risk of bias of individual studies assessed using the Newcastle–Ottawa Scale for assessing risk of bias. The maximum possible score was 9 points (63 points for the seven studies). High risk was defined as 0 to 3 points, moderate risk of bias was considered as 4 to 6 points, and, finally, a low risk of bias was assigned to studies scoring between 7 and 9 points. Each * is one point.

Studies	Sample Selection			Outcome		Risk of Bias
	Representativeness	Sample Size	Menopausal Status	Outcome Assessment	No. of Observers	
Lee et al., 2022 [12]	***	0	**	*	0	Moderate (6)
Foglio-Bonda et al., 2019 [11]	*	0	0	*	0	High (2)
Rukmini et al., 2018 [24]	*	*	**	*	0	Moderate (5)
Parakh et al., 2016 [25]	*	0	**	*	0	Moderate (4)
Bhat et al., 2010 [26]	*	0	**	*	0	Moderate (4)
Dural et al., 2006 [27]	*	0	**	*	0	Moderate (4)
Yalcin et al., 2005 [28]	*	0	**	*	0	Moderate (4)
Overall	9	1	12	7	0	Moderate (29)

**Table 5:** GRADE assessment of certainty level. This table summarizes the evidence on the association between menopause and dental caries status the prevalence of dental caries, based on the GRADE approach.

No. of Studies	Study Design	Risk of Bias	Inconsistency	Indirectness	Imprecision	Other Considerations	Certainty
Postmenopausal women—dental caries status							
7	Observational studies	Not serious ^a^	Serious ^b^	Serious	Serious ^c^	MD = 3.13 (2.12–4.15) *p* < 0.00001	⊕○○○ Very low

GRADE Working Group grades of evidence, explanations: ^a^ Detailed in Table 4: Risk of bias summary (moderate). ^b^ I^2^ = 80% (*p* < 0.0001). ^c^ 95% CI out of 0.75–1.25. Very low certainty: The true effect might be substantially different from the estimated effect.

## Data Availability

The data are public.

## Abbreviations

The following abbreviations are used in this manuscript:

DMFT	Decayed, Missing, and Filled Teeth
MD	Mean Difference
MESH	Medical Subject Headings
CI	Confidence Interval
GRADE	Grading of Recommendations Assessment, Development, and Evaluation
HRT	Hormone Replacement Therapy
